# Social apoptosis in honey bee superorganisms

**DOI:** 10.1038/srep27210

**Published:** 2016-06-06

**Authors:** Paul Page, Zheguang Lin, Ninat Buawangpong, Huoqing Zheng, Fuliang Hu, Peter Neumann, Panuwan Chantawannakul, Vincent Dietemann

**Affiliations:** 1 Agroscope, Swiss Bee Research Center, Schwarzenburgstrasse 161, 3003 Bern, Switzerland; 2 College of Animal Sciences, Zhejiang University, Yuhangtang Road 866, Hangzhou, China; 3 Bee protection laboratory, Department of Biology, Faculty of Science, Chiang Mai University, Chiang Mai, Thailand 50200; 4 Institute of Bee Health, Vetsuisse Faculty, University of Bern, Bern, Switzerland; 5 Social Insect Research Group, Department of Zoology and Entomology, University of Pretoria, Private Bag X20, Hatfield, Pretoria 0028, South Africa

## Abstract

Eusocial insect colonies form superorganisms, in which nestmates cooperate and use social immunity to combat parasites. However, social immunity may fail in case of emerging diseases. This is the case for the ectoparasitic mite *Varroa destructor*, which switched hosts from the Eastern honeybee, *Apis cerana*, to the Western honey bee, *Apis mellifera*, and currently is the greatest threat to *A. mellifera* apiculture globally. Here, we show that immature workers of the mite’s original host, *A. cerana,* are more susceptible to *V. destructor* infestations than those of its new host, thereby enabling more efficient social immunity and contributing to colony survival. This counterintuitive result shows that susceptible individuals can foster superorganism survival, offering empirical support to theoretical arguments about the adaptive value of worker suicide in social insects. Altruistic suicide of immature bees constitutes a social analogue of apoptosis, as it prevents the spread of infections by sacrificing parts of the whole organism, and unveils a novel form of transgenerational social immunity in honey bees. Taking into account the key role of susceptible immature bees in social immunity will improve breeding efforts to mitigate the unsustainably high colony losses of Western honey bees due to *V. destructor* infestations worldwide.

Honey bee, *Apis* spp., colonies can be regarded as superorganisms, in which cooperating individuals in overlapping generations support functions comparable to those of cells in a multicellular organism^1^. Such cooperation can foster the survival of parasite-infested colonies via social immunity^2^,^3^, e.g. social analogues of encapsulation^4^ or fever^5^. However, social immunity might be inefficient if parasites shift host species such as in the case of the ectoparasitic mite *Varroa destructor*, which originally infested the Eastern honey bee *A. cerana*, and now infests the Western honey bee, *A. mellifera*, at a global scale^6^. There is general consensus that this ubiquitous mite, together with the viruses it vectors^6^, is the main biotic factor threatening *A. mellifera* colony survival, because infestations usually result in colony death within 2–3 years^6^,^7^,^8^,^9^. This is due to the exponential growth of mite populations sustained by developing worker brood throughout the year and seasonal male brood^6^. In sharp contrast, infested colonies of the original host, *A. cerana*, are surviving. Among several potential resistance traits of *A. cerana*^6^, the apparent absence of mite population growth in association with worker brood is likely to be most significant^10^. Other *A. cerana* populations naturally infested with different mite species and haplotypes also appear resistant to the ubiquitous invasive *V. destructor* Korean haplotype infesting sympatric *A. mellifera* in Asia^11^. Social immunity^2^ is likely to play a major role as resistance mechanism; for instance, adult honey bee workers cooperate to detect and remove worker brood infested by this mite, thereby interrupting its reproduction^6^,^12^. The trigger of this hygienic behaviour by workers is based on signals from infested brood^13^ and/or by cues from the parasite^14^, on the ability of workers to detect this information as well as on their response thresholds^11^,^15^,^16^. The ability of *A. cerana* workers to perform hygienic behaviour towards *V. destructor*-infested brood is greater than that of the new host, *A. mellifera*^17^. However, the mechanisms underlying this major resistance trait of *A. cerana* are unknown. In particular, the crucial role of mite-infested brood in inducing this behaviour has not yet been fully investigated, since all previous studies have been performed in the presence of adult workers, thereby confounding the respective roles of signals/cues, of detection abilities and of response thresholds, which may all contribute to efficient hygienic brood removal.

Here, we took an alternative approach to address the apparent gaps in our understanding of the mechanisms underlying the principal resistance traits of the original host of *V. destructor, A. cerana*. The traditional approach to dealing with complexity is to reduce or constrain it. Here, we experimentally reduce the complexity of our system by excluding one level (adult workers), thereby allowing for the first time to distinguish between the effects of brood and adult workers in the hygienic behaviour. By monitoring the development and survival of mite-infested honey bee brood of *A. cerana* and *A. mellifera* colonies in both the absence and presence of adult workers, we separated the respective roles of the developing brood from that of adult nestmates in the resulting hygienic behaviour. We hypothesised that the brood of *A. cerana* is more susceptible compared to *A. mellifera*, thereby providing the basis for a more efficient removal behaviour by adult nestmates. A higher susceptibility of infested or wounded worker brood associated with efficient hygienic behaviour in adult workers is expected to reinforce the social immunity of a honey bee colony infested by brood parasites or pathogens and may contribute to colony survival.

## Results and Discussion

We infested 314 worker larvae from 6 colonies in one population of *A. mellifera* and from 4–5 colonies in each of three distant populations of *A. cerana*. For infestations, we used the invasive Korean haplotype of *V. destructor*^18^. Wax combs containing infested and non-infested control brood cells were placed in an incubator mimicking hive conditions for optimal brood development^19^. The cells were opened one day prior to the expected emergence date of the adult bees and the developmental stages of the individuals were recorded^20^.

**Figure 1:**
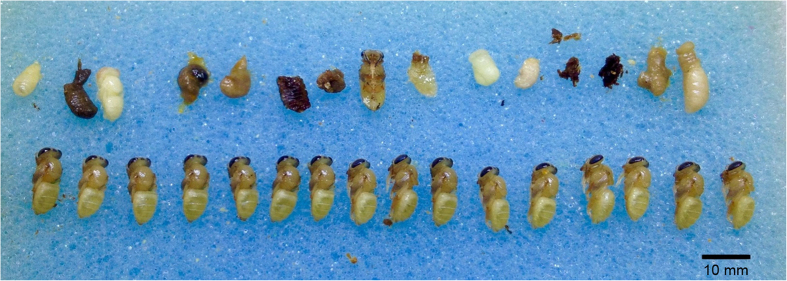
Mite-infested brood (above row) and control brood (bottom row) from an *A. cerana* colony (Phatthalung, Thailand). Individuals were removed from their cells one day before emergence was expected. Most infested individuals stopped development at the larval and prepupal stages and died, Photo by Paul Page.

**Figure 2:**
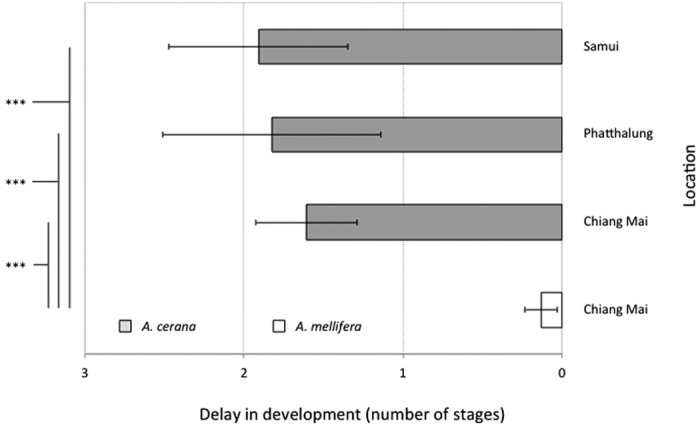
Delay in brood development calculated as the difference in number of developmental stages (x axis) separating infested brood from non-infested (control) brood in three populations of *A. cerana* and one of *A. mellifera* (y axis). The average developmental delays within were compared by using a one-way ANOVA on log-transformed values combined with a Dunnett’s *post hoc* test using *A. mellifera* as control group. Values represented are means ± 1 S.E.M. ***P < 0.001.

The data shows a striking difference in the effect of parasitism between *A. cerana* and *A. mellifera*. The development of *A. mellifera* worker brood infested by *V. destructor* was similar to the non-infested controls. In contrast, in the three *A. cerana* populations, we found a higher frequency of individuals at early developmental stages in infested worker brood than in control brood (Fig. 1; Supplementary Fig. S1). Overall, the development of infested individuals was significantly delayed in *A. cerana* compared to *A. mellifera* (one-way ANOVA on log-transformed values with a Dunnett’s *post hoc* test using *A. mellifera* as control group, df = 3, F = 7.2, p = 0.003, Fig. 2). Whenever we observed larvae or pre-pupae (earliest developmental stages) one day prior to expected emergence, most of them were decomposed and were obviously dead (Fig. 1).

**Figure 3:**
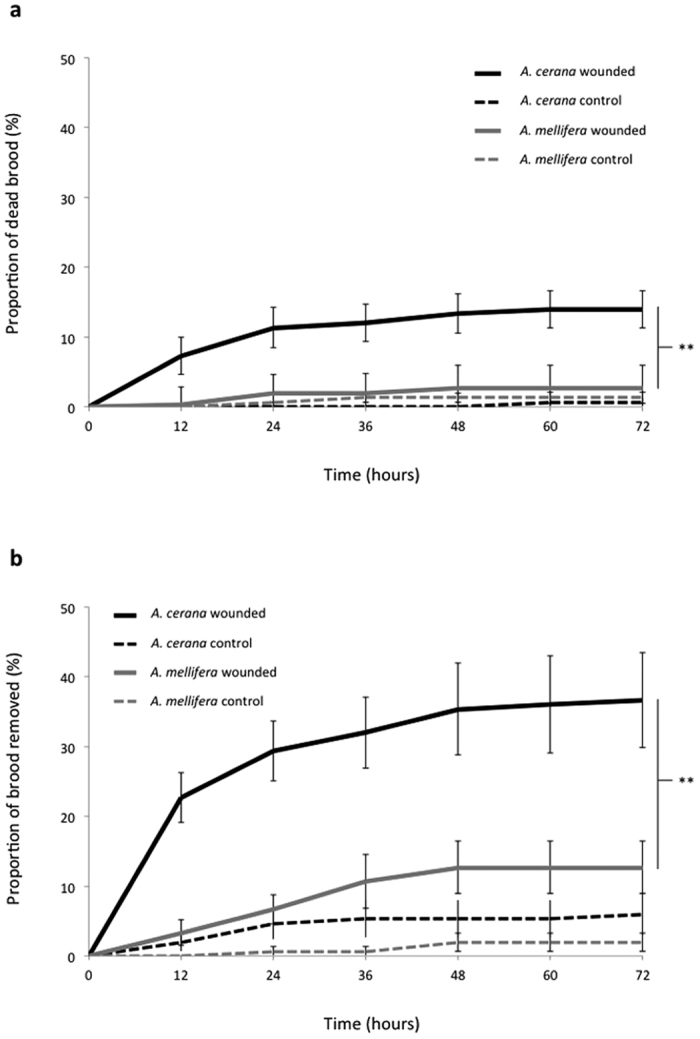
(**a)** Mortality of wounded brood in *A. cerana* and *A. mellifera* developing in an incubator. The survival status of larvae pricked with a sterile glass-pulled needle and of control larvae was monitored every 12 hours during three days; (**b**) Removal of wounded brood in *A. cerana* and *A. mellifera* colonies. Larvae pricked with a sterile glass-pulled needle and controls were exposed to workers and their removal via hygienic behaviour was monitored every 12 hours during three days. Brood mortality and removal rates were compared with log-rank Mantel-tests. Values are means ± 1 S.E.M. **P < 0.01.

In the presence of workers, and even when assuming an equal ability of both honey bee species to detect signals triggering hygienic behaviour as well as equal response thresholds towards them, this observed difference in brood susceptibility is likely to elicit an earlier and more frequent production of such signals, ultimately leading to more efficient removal behaviour and overall enhanced social immunity. In order to test this hypothesis, we subjected larvae of both species to a benign wounding and either placed them in an incubator to monitor their development or back into their colony of origin. *A. cerana* brood died more quickly and in greater proportions than *A. mellifera* in incubator conditions (Log-rank Mantel test, df = 1, Chi^2^ = 12.8, p < 0.001, Fig. 3a; Supplementary Fig. S2) and in colonies, *A. cerana* workers removed the wounded brood significantly more and faster than *A. mellifera* (Log-rank Mantel test, df = 1, Chi^2^ = 67.8, p < 0.001, Fig. 3b). These results indicate that *A. cerana* brood was affected faster and to a higher degree by wounding than *A. mellifera* and that wounded brood could be detected by workers for a more efficient hygienic removal. The higher proportion of brood removed by the workers in the colonies compared to our estimate of brood death in the incubator (Fig. 3) is likely due to the sensitivity of workers to dysfunctional individuals that could have survived in incubator conditions. Nonetheless, a benign wounding of *A. cerana* larvae appears to be sufficient to yield a reaction analogous to apoptosis in multicellular organisms. Given the use of a sterile pin for the pricking, these results also allow the exclusion of secondary pathogens commonly carried by *V. destructor*^6^ in the mortality observed.

Hence, the significantly higher susceptibility of mite-infested brood of the original host of *V. destructor* leads to more efficient hygienic behaviour, thereby providing a basis for honey bee colony survival to parasitism and constituting an additional resistance trait of the original host of this parasite. Our results provide a most parsimonious explanation for the striking differences in the impact of infestations by the invasive Korean haplotype of *V. destructor* mites between the Western and the Eastern honey bee species. Brood susceptibility could also contribute to colony survival to *V. destructor* mite infestations in naturally resistant *A. mellifera* populations^6^,^21^,^22^,^23^,^24^.

Our counterintuitive result shows that susceptible individuals can benefit the superorganism, which goes against the common assumption that ‘strong’ elements of a social entity are required to ensure group survival. We here show that ‘weak’ elements can contribute to the strength of a social ensemble. This provides empirical support to theories on the adaptive value of suicide or high death rate in social insects^25^,^26^, thereby constituting a social analogue of apoptosis, a mechanism preventing the spread of infections by sacrificing parts of the whole organism^27^,^28^. Given the high number of individuals in a honey bee colony, some individuals can be (and are commonly) sacrificed without compromising the survival of the group^29^ and even promote colony survival by hindering further infestations^30^. Such altruistic suicide of immature individuals is a novel form of transgenerational social immunity in honey bees. Irrespective of the actual pathogen, the observed social apoptosis is most likely a fundamental defence mechanism of social insect colonies to combat diseases.

Mitigating the elevated losses of managed Western honey bee colonies, *A. mellifera*, experienced globally^8^ is crucial to ensure the sustainability of their valuable pollination services towards agriculture^31^ and ecosystem functioning^32^. If *A. mellifera* populations are to show variation in brood damage linked to *V. destructor* infestation or other diseases, a breeding concept based on brood susceptibility could be implemented. This might increase the efficiency of breeding programs and thus help alleviate the global losses of managed honey bees due to this parasitic mite and other brood diseases.

## Materials and Methods

### Infestation experiments

#### Mite collections and honey bee colonies

Adult *V. destructor* female mites were collected from drone and worker brood of *A. mellifera* colonies. We used the same colonies as mite sources to infest both species at each location. Batches of 10–20 collected mites were kept on 15–20 *A. mellifera* adult workers in plastic cages for at least two days, thereby mimicking the wandering stage of mites prior to reproduction in honey bee brood cells^6^. The invasive K1 (Korean) *V. destructor* haplotype was confirmed on a mite subsample (N = 146) using standard methods^20^ by comparing mitochondrial DNA sequences to references deposited in GenBank (*V. destructor* Cox-1 gene 458 bp fragment; Korean haplotype, accession number AF106899.1)^18^. Infestation experiments were performed in six colonies of *A. mellifera* in Chiang Mai (Northern Thailand; N 18°48′13″ and E 98°57′22″, *A. mellifera* can only survive in the northern part of the country) and on five, five and four colonies of *A. cerana* in Chiang Mai, Phatthalung (Southern Peninsular Thailand; Khuan Khanun, N 7°44′57″ and E 100°00′03″) and Samui island (N 9°32′05″ and E 99°58′28″), respectively.

#### Brood comb preparation and infestations

*V. destructor* mites reproduce on honey bee brood developing within the wax cells of host nests. They enter these cells just before they are sealed with a wax cap by workers^6^. In *A. mellifera* combs, we mapped the positions of cells containing uncapped larvae at the appropriate stage (L5 larvae) on transparent sheets and reintroduced the comb into its colony of origin for natural capping by the workers to occur. Experimental cells were then selected among those that were capped within 6 hours and subsequently used for infestations^20^. Such infestations do not limit mite reproduction^33^,^34^. We partially opened the capping of selected cells using a razor blade edge and assigned one of two treatments: (1) infested cells: a single adult *V. destructor* female mite was introduced into the cell by using a fine paint brush and (2) control cells: the opening was only lightly brushed without introducing a mite. In both cases, the cell capping was then resealed.

In *A. cerana*, natural wax combs are not built on wooden frames so they cannot be placed back into their colony of origin to be capped naturally. Thus, the cells used for these experiments were identified based on a visual assessment of larval developmental stage and when possible of partial capping of the cells. Infestation (1) or sham treatment (2) was performed as above for *A. mellifera*, but the cells were capped with a layer of paper tissue. This layer of paper was sealed to the cell walls with the use of a pre-heated nail head.

For each colony, we used approximately the same number of infested and control cells, which varied from 8–45 and 9–46 respectively, depending on the number of L5 larvae available at the beginning of each infestation experiment. Once the infestations were performed, we cut out the portion of comb with the infested cells and suspended it in an incubator at developing brood conditions, i.e. 34.5 °C and 70–90% RH. Whenever field conditions did not allow us an access to a laboratory incubator, we used a portable incubator connected to a 12 V battery and yielding identical temperature and humidity conditions in order to store the combs. *Apis cerana* and *A. mellifera* workers display the same developmental periods before capping: 3 days from egg to larva followed by 6 days of molting larval instars (L1 to L5). After capping occurs, however, their preimaginal stages differ by one day, as *A. mellifera* workers need 12 days of pupation to fully develop into an adult, while *A. cerana* workers only require 11 days until bee emergence^35^. We thus opened the experimental cells one day before the expected emergence date and reported the developmental stage of the opened brood as well as its survival status. We recorded the developmental data as follows: larva, pre-pupa, white-eyed, pink-eyed and purple-eyed pupae were considered to be immature stages of development (stages 1–5), whereas yellow-thorax, grey-winged, grey-thorax, grey-abdomen pupae and adults were defined as mature stage (stage 6) based on the developmental pattern observed in the control at the time of cell opening.

#### Data and statistical analyses

We used data from cells containing brood that was not affected by chalkbrood infection, by wax moths or by involuntary crushing of the comb. In addition, we only considered artificially infested cells that showed a clear sign of infestation, i.e. the occurrence of feces or the presence of a *V. destructor* mite in the cell, and that were not naturally infested by either *Tropilaelaps* spp. or local *Varroa* spp. mites, prior to the experiment.

We ranked each individual from 1 (larva) to 6 (mature), depending on its developmental stage at cell opening. Within each replicate, we calculated an average developmental stage for infested and control cells separately and subtracted the former from the latter, in order to obtain a mean developmental delay per colony. All residuals were normally distributed within populations and the variance was homogeneous between populations. We then compared the average developmental delays within each species and each location by using a one-way ANOVA on log-transformed values combined with a Dunnett’s *post hoc* test and *A. mellifera* as the control group. Statistical calculations were performed using Systat software (version 13).

Further analyses were performed with generalized linear models (GLMs) applied to the same developmental stage dataset with colony as random factor and using the *lmer* function from the package *lme4*^36^. Response variables were the frequencies of control and infested individuals at each stage in each colony. They were modelled with Poisson error distributions. Statistical tests were performed in R software (R version 3.2.2)^37^ and are summarized in the Supplementary Table S1.

### Larvae wounding experiments

#### Honey bee colonies and brood comb preparations

We used 5 colonies of *Apis mellifera* and 5 of *Apis cerana* kept in Langstroth hives in an apiary at Zhejiang University, Hangzhou (China) for the larval wounding experiments. On the combs of both species, we identified cells by mapping brood at the L5 stage, as described above. We then replaced the comb into the colony for capping by the workers to occur and identified the capped cells 6 hours after the mapping. We used a fine pulled-glass capillary with a diameter similar to that of the *V. destructor* mite chelicerae (Ø = 50 μm) to wound the larvae by pricking, simulating the wound induced by *V. destructor* mites feeding on the bee larvae^38^,^39^. We inflicted wounding by pressing the tip of the capillary against the cuticle until it gave way. We sterilized the needle with ethanol before each use and replaced it anew for each colony tested. Two experiments were conducted in each species in order to quantify the susceptibility of larvae towards wounding, firstly in the absence of workers and secondly in their presence to compare the removal of wounded brood by workers, i.e. hygienic behaviour.

(1) To measure the susceptibility of wounded larvae, we pricked 30 freshly capped larvae and instead of resealing the wax capping, we removed it and closed the cell with a transparent gelatin cap, allowing for the observation of brood development^40^. As a control, we opened 15 freshly capped cells and sealed them with a gelatin cap without pricking the larvae (controls for the effect of pricking) and we left 15 cells non-manipulated (controls for the effect of gelatin cap on larva survival). We then placed the comb into an incubator at developing brood conditions, i.e. 34.5 °C and 70% RH, and checked the experimental cells every 12 hours during the next three days. At every check, we reported the survival status of the brood in the cells. We considered a larva dead when it turned black and deflated or when it did not stretch in the cell as is typical for the transition to pre-pupal stage. We opened the non-manipulated cells after 72 hours to identify the final state of larvae/pre-pupae.

(2) To measure the hygienic removal of wounded larvae, we opened 30 freshly capped cells and gently pricked the larvae inside of them. As a control, we opened 15 cells without pricking the larvae (positive controls). Wax caps of cells with pricked and non-pricked larvae were then resealed. As a control for the effect of cell opening on removal by workers, we mapped an additional 15 cells that we did not manipulate (negative controls). The comb was then replaced into its original colony and the experimental cells were checked every 12 hours during the next 3 days. At every comb check, we reported the number of mapped brood cells that had been cleaned out by the workers of each species during an interval, i.e. brood removal.

#### Data and statistical analyses

(1) To assess susceptibility to wounding, we counted the dead and surviving larvae in each species and at each time interval. (2) To compare hygienic removal of wounded brood, we counted the number of wounded brood removed by the workers of each species at each time interval. As the respective mortality and removal rates of the different controls were not significantly different for either experiment (log-rank Mantel test; susceptibility: *A. mellifera*, df = 1, Chi^2^ = 0.34, p = 0.56; *A. cerana*, df = 1, Chi^2^ = 0.0, p = 0.99; hygienic removal: *A. mellifera*, df = 1, Chi^2^ = 2.0, p = 0.16; *A. cerana*, df = 1, Chi^2^ = 1.0, p = 0.32), these data were pooled. The validity of our assays was confirmed by the control mortality being below a 15% threshold^41^. We then compared the mortality rates and wounded brood removal rates between bee species by means of log-rank Mantel tests.

## Additional Information

**How to cite this article**: Page, P. *et al*. Social apoptosis in honey bee superorganisms. *Sci. Rep.* **6**, 27210; doi: 10.1038/srep27210 (2016).

## Supplementary Material

Supplementary Information

## References

[b1] Southwick E. E. & Moritz R. F. Bees as Superorganisms: an Evolutionary Reality. Springer-Verlag, New York (1992).

[b2] Evans J. D. & Spivak M. Socialized medicine: Individual and communal disease barriers in honey bees. J. Invert. Pathol. 103, S62–S72 (2010). 10.1016/j.jip.2009.06.019 19909975

[b3] Meunier J. Social immunity and the evolution of group living in insects. Phil. Trans. R. Soc. B. 370, 20140102 (2015). 25870389 10.1098/rstb.2014.0102 PMC4410369

[b4] Neumann P. . Social encapsulation of beetle parasites by Cape honeybee colonies (*Apis mellifera capensis* Esch.). Naturwissenschaften. 88, 214–216 (2001). 11482434 10.1007/s001140100224

[b5] Starks P. T., Blackie C. A. & Seeley T. D. Fever in honeybee colonies. Naturwissenschaften. 87, 229–231 (2000). 10883439 10.1007/s001140050709

[b6] Rosenkranz P., Aumeier P. & Ziegelmann B. Biology and control of *Varroa destructor*. J. Invert. Pathol. 103, S96–S119 (2010). 10.1016/j.jip.2009.07.016 19909970

[b7] Dietemann V. . *Varroa destructor*: research avenues towards sustainable control. J. Apicult. Res. 51, 125–132 (2012).

[b8] Neumann P. & Carreck N. L. Honey bee colony losses. J. Apicult. Res. 49, 1–6 (2010).

[b9] Eisenstein M. Pesticides: seeking answers amid a toxic debate. Nature 521, S52–S55 (2015). 25992672 10.1038/521S52a

[b10] Rath W. Co-adaptation of *Apis cerana* Fabr. and *Varroa jacobsoni* Oud. Apidologie 30, 97–110 (1999).

[b11] Navajas M. . Differential gene expression of the honey bee *Apis mellifera* associated with *Varroa destructor* infection. BMC Genomics 9, 301 (2008). 18578863 10.1186/1471-2164-9-301 PMC2447852

[b12] Arathi H. S. & Spivak M. Influence of colony genotypic composition on the performance of hygienic behaviour in the honeybee, *Apis mellifera* L. Anim. Behav. 62, 57–66 (2001).

[b13] Nazzi F., Della Vedova G. & d’Agaro M. A semiochemical from brood cells infested by *Varroa destructor* triggers hygienic behaviour in *Apis mellifera*. Apidologie 35, 65–70 (2004).

[b14] Rosenkranz P., Tewarson N. C., Singh A. & Engels W. Differential hygienic behaviour towards *Varroa jacobsoni* in capped worker brood of *Apis cerana* depends on alien scent adhering to the mites. J. Apicult. Res. 32, 89–93 (1993).

[b15] Chakroborty N. K., Bienefeld K. & Menzel R. Odor learning and odor discrimination of bees selected for enhanced hygienic behavior. Apidologie. 46, 499–514 (2015).

[b16] Wilson-Rich N., Spivak M., Fefferman N. F. & Starks P. T. Genetic, individual and group facilitation of disease resistance in insect societies. Annu. Rev. Entomol. 54, 405–423 (2009). 18793100 10.1146/annurev.ento.53.103106.093301

[b17] Peng Y. S. C., Fang Y., Xu S. & Ge L. The resistance mechanism of the Asian honey bee, *Apis cerana* Fabr., to an ectoparasitic mite, *Varroa jacobsoni* Oudemans. J. Invert. Pathol. 49, 54–60 (1987).

[b18] Anderson D. L. & Trueman J. W. H. *Varroa jacobsoni* (Acari: Varroidae) is more than one species. Exp. Appl. Acarol. 24, 165–189 (2000). 11108385 10.1023/a:1006456720416

[b19] Williams G. R. . Standard methods for maintaining adult *Apis mellifera* in cages under *in vitro* laboratory conditions. J. Apicult. Res. 52, 1–36 (2013).

[b20] Dietemann V. . Standard methods for varroa research. J. Apicult. Res. 52, 1–54 (2013).

[b21] Le Conte Y. . Honey bee colonies that have survived *Varroa destructor*. Apidologie. 38, 566–572 (2007).

[b22] Fries I. & Bommarco R. Possible host-parasite adaptations in honey bees infested by *Varroa destructor* mites. Apidologie. 38, 525–533 (2007).

[b23] Seeley T. D. Honey bees of the Arnot Forest: a population of feral colonies persisting with *Varroa destructor* in the northeastern United States. Apidologie. 38, 19–29 (2007).

[b24] Strauss U., Pirk C. W., Dietemann V., Crewe R. M. & Human H. Infestation rates of *Varroa destructor* and *Braula coeca* in the savannah honey bee (*Apis mellifera scutellata*). J. Apicult. Res. 53, 475–477 (2014).

[b25] Smith Trail D. R. Behavioral interactions between parasites and hosts: host suicide and the evolution of complex life cycles. Am. Nat. 116, 77–91 (1980).

[b26] Betti M. I., Wahl L. M. & Zamir M. Effects of infection on honey bee population dynamics: a model. PLoS ONE 9, e110237 (2014). 25329468 10.1371/journal.pone.0110237 PMC4199628

[b27] Elmore S. Apoptosis: A review of programmed cell death. Toxicol. Pathol. 35, 495–516 (2007). 17562483 10.1080/01926230701320337 PMC2117903

[b28] Fuchs Y. & Steller H. Programmed cell death in animal development and disease. Cell 147, 742–758 (2011). 22078876 10.1016/j.cell.2011.10.033 PMC4511103

[b29] Shorter J. R. & Rueppell O. A review on self-destructive defense behaviors in social insects. Insect. Soc. 59, 1–10 (2012).

[b30] Rueppell O., Hayworth M. K. & N. P. Ross Altruistic self-removal of health-compromised honey bee workers from their hive. J. Evol. Biol. 23, 1538–1546 (2010). 20500363 10.1111/j.1420-9101.2010.02022.x

[b31] Breeze T. D. . Agricultural policies exacerbate honeybee pollination service supply-demand mismatches across Europe. PLoS ONE 9, e82996 (2014). 24421873 10.1371/journal.pone.0082996 PMC3885438

[b32] Potts S. G. . Global pollinator declines: trends, impacts and drivers. Trends Ecol. Evol. 25, 345–353. 20188434 10.1016/j.tree.2010.01.007

[b33] Garrido C. & Rosenkranz P. The reproductive program of female *Varroa destructor* mites is triggered by its host, *Apis mellifera*. Exp. Appl. Acarol. 31, 269–273 (2003). 14974691 10.1023/b:appa.0000010386.10686.9f

[b34] Frey E., Odemer R., Blum T. & Rosenkranz P. Activation and interruption of the reproduction of *Varroa destructor* is triggered by host signals (*Apis mellifera*). J. Invert. Pathol. 113, 56–62 (2013). 10.1016/j.jip.2013.01.007 23376006

[b35] Oldroyd B. P. & Wongsiri S. Asian Honey Bees: Biology, Conservation and Human Interactions. Harvard University Press, Cambridge (2006).

[b36] Bates D., Maechler M., Bolker B. & Walker S. Fitting Linear Mixed-Effects Models Using lme4. J. Stat. Softw. 67, 1–48 (2015).

[b37] R Development Core Team. R: A language and environment for statistical computing. R Foundation for Statistical Computing, Vienna, Austria. ISBN 3-900051-07-0, http://www.R-project.org (2015).

[b38] Kanbar G. & Engels W. Ultrastructure and bacterial infection of wounds in honey bee (*Apis mellifera*) pupae punctured by *Varroa* mites. Parasitol. Res. 90, 349–354 (2003). 12684884 10.1007/s00436-003-0827-4

[b39] Herrmann M., Kanbar G. & Engels W. Survival of honey bee (*Apis mellifera*) pupae after trypan blue staining of wounds caused by *Varroa destructor* mites or artificial perforation. Apidologie 36, 107–111 (2005).

[b40] de Guzman L. I., Khongphinitbunjong K., Rinderer T. E., Tarver M. R. & Frake A. M. A laboratory technique to study the effects of *Varroa destructor* and viruses on developing worker honey bees. J. Apic. Res. 52, 262–264 (2013).

[b41] Alix A. . Environmental risk assessment scheme for plant protection products. EPPO Bulletin, chapter 10: Honeybees – Proposed scheme. Julius-Kühn-Archiv. 423, 27–33 (2010).

