# Supplementary material for: Social apoptosis in honey bee superorganisms
Source: Sci Rep. 2016 Jun 6;6:27210. doi: 10.1038/srep27210 (PMC4893659; doi:10.1038/srep27210)
Supplement: Supplementary Information [file srep27210-s1.pdf]

Social apoptosis in honey bee superorganisms

Authors

Paul Page[1]*, Zheguang Lin[2]*, Ninat Buawangpong[3]*, Huoqing Zheng[2,#], Fuliang Hu[2], Peter Neumann[3,4,5], Panuwan Chantawannakul[3,#], Vincent Dietemann[1,5]

Supplementary information includes:

Figure S1

Table S1

Figure S2

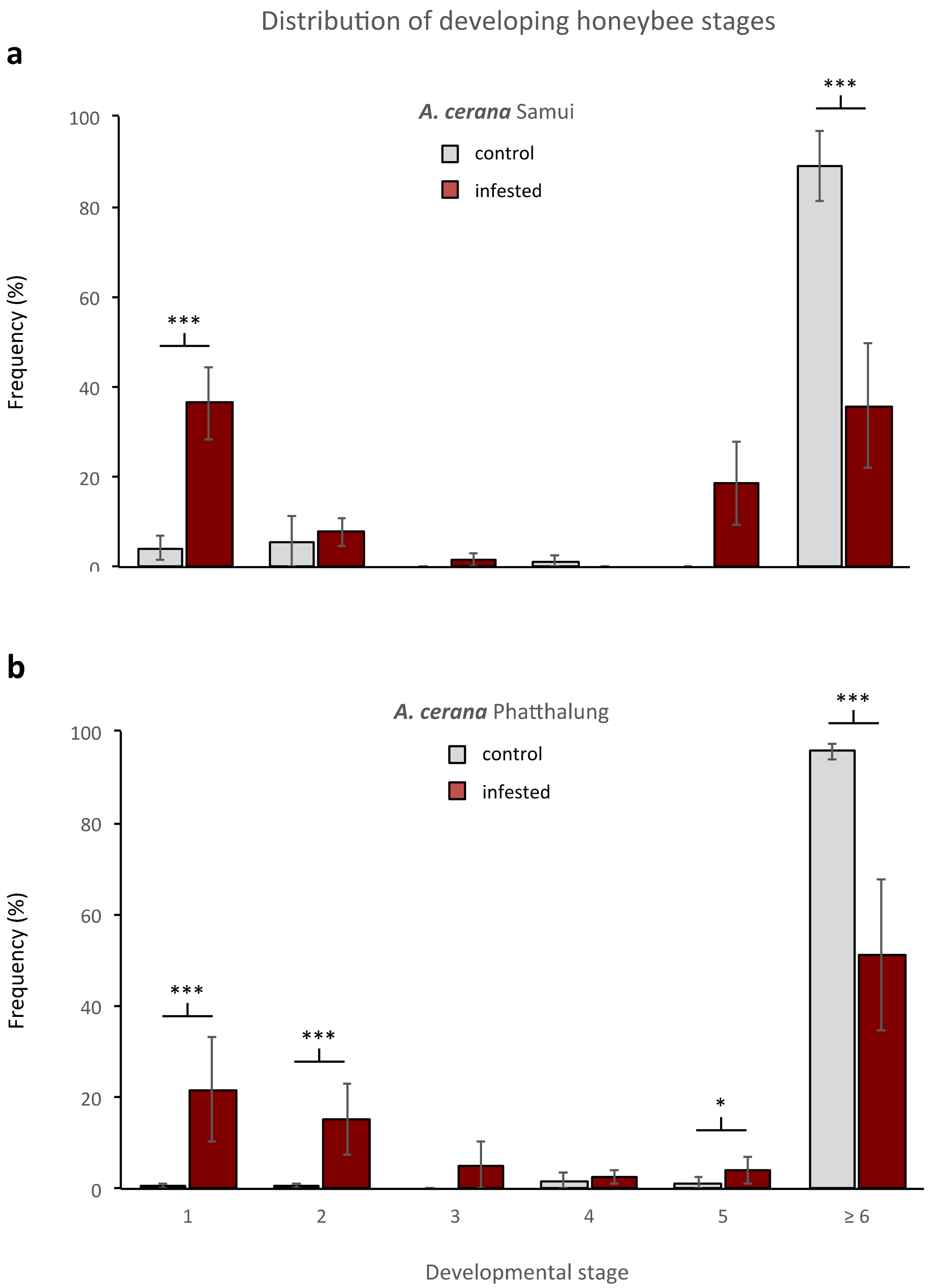

Distribution of developing honeybee stages
a
A. cerana Samui
control
infested
Frequency (%)
100
80
60
40
20
0
***
***
b
A. cerana Phatthalung
control
infested
Frequency (%)
100
80
60
40
20
0
***
***
*
***
1
2
3
4
5
≥ 6
Developmental stage

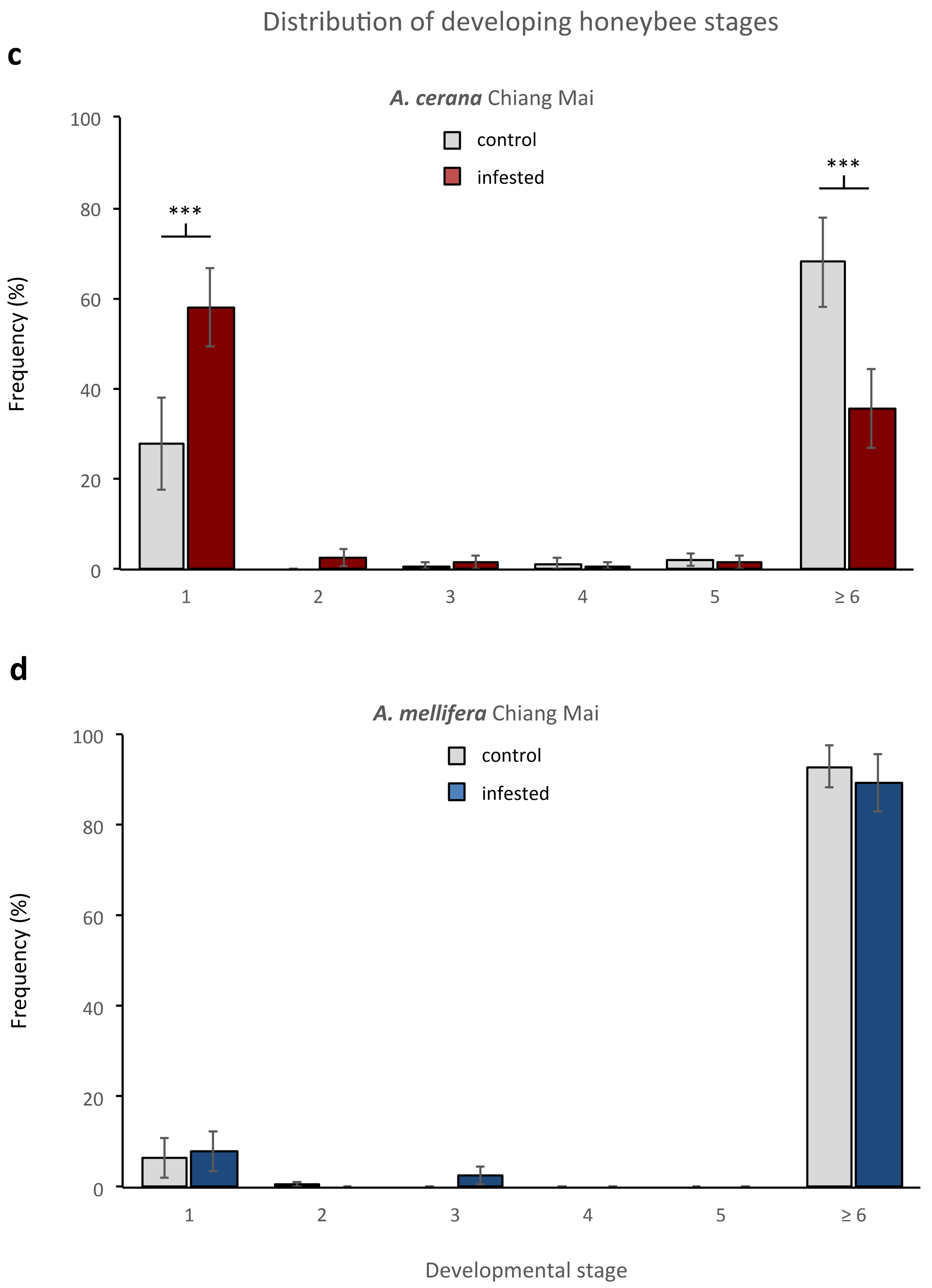

Supplementary Figure S1. Frequency distributions of developing worker brood in the cross-fostering experiments. Infested and non-infested (control) brood in three populations of *A. cerana* (**a-c**) and in one population of *A. mellifera* (**d**). Stages 1-6 represent respectively larvae, pre-pupae, white-eyed, pink-eyed and purple-eyed pupae and mature stages. Values are means ± 1 S.E.M. *** P < 0.001 and * *P* < 0.05. Statistical outputs are summarised in the Supplementary Table 1.

Supplementary Table S1. Statistical output of the GLMs performed on the frequency distributions of developing worker brood in four *Apis* populations in the infestation experiments performed in Thailand. P-values indicate significance of the differences between control and infested individuals within each developmental stage (1-6).

Location	Species	Developmental stage	Estimate	Standard Error	z-value	Pr(>|z|)	Significance
Samui	*A. cerana*	1	2.21	0.26	8.40	> 0.001	***
		2	0.34	0.28	1.23	0.22	
		3	17.71	1734.00	0.01	0.99	
		4	-17.53	1733.93	-0.01	0.99	
		5	20.23	1734.00	0.01	0.99	
		6	-0.91	0.10	-9.17	> 0.001	***
Phatthalung	*A. cerana*	1	3.59	0.59	6.14	> 0.001	***
		2	3.23	0.59	5.49	> 0.001	***
		3	16.95	570.53	0.03	0.98	
		4	0.49	0.45	1.08	0.28	
		5	1.05	0.44	2.39	0.02	*
		6	-0.63	0.08	-8.10	> 0.001	***
Chiang Mai	*A. cerana*	1	0.74	0.10	7.13	> 0.001	***
		2	17.33	940.65	0.02	0.99	
		3	0.69	0.61	1.13	0.26	
		4	-0.56	0.63	-0.89	0.37	
		5	-0.54	0.48	-1.13	0.26	
		6	-0.64	0.09	-6.95	> 0.001	***
Chiang Mai	*A. mellifera*	1	0.18	0.21	0.85	0.39	
		2	-17.61	2334.17	-0.01	0.99	
		3	19.34	2334.00	0.01	0.99	
		4	0.00	3301.00	0.00	1.00	
		5	0.00	3301.00	0.00	1.00	
		6	-0.04	0.06	-0.64	0.53	

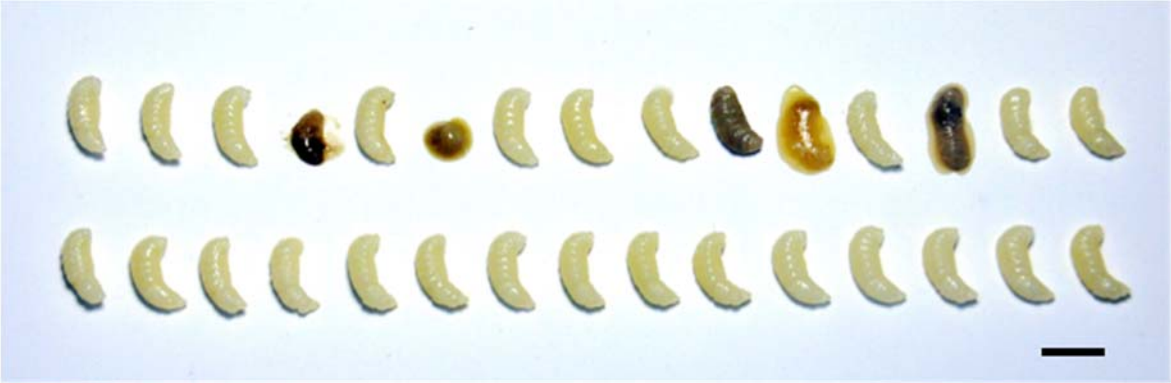

Supplementary Figure S2. Appearance of wounded (above row) and control (bottom row) worker larvae from an *A. cerana* colony in Hangzhou (China). The cells of freshly capped worker larvae were opened on one comb per colony and individuals were either benignly wounded with a sterile pulled-glass pin or left untouched. The experimental cells were then sealed with see-through gelatine caps[38] and placed in an incubator at 34.5 °C and 70% RH. The status of the developing brood was assessed every 12 hours for three days. After the last assessment the larvae were extracted from the cells to be photographed. The scale bar represents 10mm.
